# Metabolomic characteristics of cholesterol-induced non-obese nonalcoholic fatty liver disease in mice

**DOI:** 10.1038/s41598-017-05040-6

**Published:** 2017-07-21

**Authors:** Lan N. Tu, Megan R. Showalter, Tomas Cajka, Sili Fan, Viju V. Pillai, Oliver Fiehn, Vimal Selvaraj

**Affiliations:** 1 Department of Animal Science, College of Agriculture and Life Sciences, Cornell University, Ithaca, NY 14853 USA; 2 West Coast Metabolomics Center, University of California Davis Genome Center, Davis, CA 95616 USA; 3 Biochemistry Department, King Abdulaziz University, Jeddah, Saudi Arabia

## Abstract

Nonalcoholic fatty liver disease (NAFLD) in non-obese patients remains a clinical condition with unclear etiology and pathogenesis. Using a metabolomics approach in a mouse model that recapitulates almost all the characteristic features of non-obese NAFLD, we aimed to advance mechanistic understanding of this disorder. Mice fed high fat, high cholesterol, cholate (HFHCC) diet for three weeks consistently developed hepatic pathology similar to NAFLD and nonalcoholic steatohepatitis (NASH) without changes to body weight or fat pad weights. Gas- and liquid chromatography/mass spectrometry-based profiling of lipidomic and primary metabolism changes in the liver and plasma revealed that systemic mechanisms leading to steatosis and hepatitis in this non-obese NAFLD model were driven by a combination of effects directed by elevated free cholesterol, cholesterol esters and cholic acid, and associated changes to metabolism of sphingomyelins and phosphatidylcholines. These results demonstrate that mechanisms underlying cholesterol-induced non-obese NAFLD are distinct from NAFLD occurring as a consequence of metabolic syndrome. In addition, this investigation provides one of the first metabolite reference profiles for interpreting effects of dietary and hepatic cholesterol in human non-obese NAFLD/NASH patients.

## Introduction

Nonalcoholic fatty liver disease (NAFLD) is the most common condition leading to chronic liver diseases, representing approximately 75% of cases in the US^1^. NAFLD first manifests as simple steatosis, or abnormal lipid accumulations in the liver. Some patients with NAFLD progress to nonalcoholic steatohepatitis (NASH), a condition characterized by hepatocyte injury, hepatic inflammation in addition to the steatosis. Advanced NASH contributes to fibrosis and may progress to cirrhosis in extreme cases. NAFLD naturally develops in the absence of alcohol abuse and the exact pathogenesis remains unknown. Moreover, NAFLD progressing to NASH currently has no effective treatment and therefore is one of the most common indications that necessitate liver transplantation^2^.

The prevalence of NAFLD is highly underestimated because it often presents with minor to no symptoms in patients at the early stages. Epidemiological studies based on clinical cases have established that prevalence of NAFLD is highest in populations with metabolic syndrome, which includes obesity, insulin resistance, hypertension, hypertriglyceridemia and low HDL levels^3, 4^. In recent years, specific screening for NAFLD in high-risk obese patients, especially those undergoing bariatric surgery, has revealed an alarming rate of asymptomatic NAFLD^5, 6^. Therefore, there has been significant focus on studying the pathophysiology of NAFLD associated with metabolic syndrome in both human and rodent models^7, 8^. Although obesity is unmistakably a predisposition to NAFLD, it is also understood that there exists a form of NAFLD in non-obese or lean subjects^9–11^.

The incidence of asymptomatic NAFLD in lean subjects has been uncovered in several unintentional assessments across distinct ethnicities. For example, as early as in 1977, analysis of liver histology obtained from 503 automobile crash victims in Denmark revealed a 24% rate of fatty liver in non-obese individuals^12^. Similarly, in a screen for healthy liver donors in the US, approximately 20% had to be excluded because of surprisingly high steatosis^13^. In Japan, a study of 39,151 individuals identified that 30.3% of the subjects had NAFLD, of which half were lean with BMI less than 25 kg/m^2^ ^14^. Another study in the US examining 11,613 individuals identified that 7.39% of lean individuals had NAFLD and 0.10% had NASH^15^. In India, a study investigating 1,168 participants reported that 48% of NAFLD cases are in non-obese patients^16^; in another study, a rural population with low prevalence of NAFLD indicated that 75% of cases had BMI less than 25 kg/m^2^ ^17^. Across these studies, non-obese NALFD patients were also non-diabetic^9, 16, 18^. Recently, it was also uncovered that long-term prognosis of non-obese NAFLD patients was worse than obese NAFLD patients with an overall higher mortality rate even when presenting a healthier metabolic profile^19^. Despite these statistics, there has been a dearth of studies attempting to understand the risk factors and pathophysiology of NAFLD in non-obese patients. Distinct characteristics of non-obese NAFLD, particularly the non-metabolic syndrome entities are issues that remains unsolved^11^.

Providing an insight into etiology, investigations on non-obese patients with NAFLD have implicated an association between dietary cholesterol and NAFLD/NASH^18, 20, 21^. Albeit understudied at the moment in NAFLD research, the relationship between cholesterol and steatosis similar to NAFLD/NASH has been long established in studies examining murine models fed a high fat, high cholesterol, cholate-containing diet (HFHCC diet; 1.25% cholesterol, 0.5% cholic acid and 15% fat^22^, originally formulated to induce atherosclerosis)^23–26^. In these mice consuming the HFHCC diet, lipid accumulation in the liver could be detected in as early as 2 days^25^, and analyses of liver histology and gene expression have demonstrated that HFHCC diet-induced NAFLD progresses from simple hepatic steatosis to NASH with pathologic damage, macrophage infiltration and inflammation in a short period of 3 weeks^23, 24, 26^. This is much earlier than the onset of atherosclerotic lesions in aortic walls after HFHCC diet that takes about 12–14 weeks^22^. Liver pathogenesis leading to NAFLD/NASH after HFHCC diet is facilitated by oxidative stress and sensitization to steatohepatitis brought about by hepatic free cholesterol (FC)^26, 27^. Moreover, long term feeding (9–12 weeks) of the HFHCC diet could induce hepatic fibrosis^26, 28^, which makes it a unique model that recapitulates all 3 stages of NAFLD without significant weight gain. The HFHCC diet-fed mice develop hypercholesterolemia, but they are insulin sensitive, not obese and plasma triglyceride levels are not elevated^26^. These characteristics are similar to human non-obese NAFLD, but are not achievable with current genetic and dietary mouse models that only focus on obese NAFLD^7, 8^. Therefore, the synergistic interactions between dietary cholesterol and fat in mice fed HFHCC diet could simulate non-obese NAFLD/NASH in patients.

In order to examine the mechanism of dietary cholesterol as a mediator in the pathogenesis of NAFLD/NASH, we profile systemic changes comparing the liver and plasma metabolomes in mice fed HFHCC diet. Characteristic changes to different metabolites documented for the first time in this study provide a foundation and advance understanding of the distinct pathophysiology associated with non-obese NAFLD/NASH.

## Materials and Methods

### Mice, diets and reagents

Male and female C57BL/6J mice at 8 weeks of age were used in the experiment in two groups (n = 6–8/group). The control group was fed a regular chow diet (Teklad 2919) that has 22% kcal from fat (9% of diet) and does not contain cholesterol or sodium cholate. The experimental group was fed the HFHCC diet (Teklad TD.90221) that contains 37.1% kcal from fat (15.8% of diet, approximately half from cocoa butter), and contains 1.25% cholesterol and 0.5% sodium cholate. Both diets were purchased sterile (irradiated) and stored as per recommendations from the manufacturer. The two groups were fed their respective diets for a 3-week period; feed provided to mice was changed every 3 days. Mice were then euthanized for collection of plasma and liver tissues. Samples were snap frozen in liquid nitrogen and stored at −80 °C until analysis. Mice were maintained and all experiments were performed in accordance with the National Institute of Health Guide for the Care and Use of Laboratory Animals. The Institutional Animal Care and Use Committee of Cornell University approved all the experiments described in this manuscript. All reagents were purchased from Sigma-Aldrich unless otherwise indicated.

### Histology, histochemistry and immunohistochemistry

Tissues were fixed with 4% formaldehyde, embedded in paraffin blocks and thin sections (4 μm) were prepared. For hematoxylin and eosin staining, sections were stained as previously described^29^. For collagen staining, sections were stained using Trichrome stain kit (Sigma) according to the manufacturer’s protocol. Images were acquired using ICC50HD camera (Leica).

Unfixed tissues were embedded frozen in optimum cutting temperature compound (Tissue-Tek) and cryostat sections (9 μm) were prepared on glass slides. For neutral lipid staining, sections were incubated with Oil Red O (Matheson Coleman & Bell) in 60% isopropyl alcohol for 30 min. Slides were then washed with distilled water and mounted using glycerol jelly. Images were acquired using ICC50HD camera (Leica). For immunohistochemistry, frozen sections were fixed with 100% acetone for 10 mins and blocked with 3% bovine serum albumin in phosphate buffer for 1 hr at room temperature. Sections were then incubated with rat monoclonal anti-CD45 (BioRad MCA1031G, dilution 1:200) overnight at 4 °C. After washing, sections were incubated with goat anti-rat IgG, Texas Red (Invitrogen T-6392, dilution 1:200) and mounted with Prolong^®^ Gold antifade mountant containing DAPI (ThermoFisher). Images were acquired using a Meta 510 confocal scope (Zeiss).

### Sample processing and preparation

Liver tissues (6 mg each) were homogenized using a mechanical disrupter (Geno/Grinder^®^). Tissue homogenates and plasma (20 uL) were extracted by adding 225 μl of cold methanol containing an internal standard mixture [lysophosphatidylethanolamine LPE(17:1), lysophosphatidylcholine LPC(17:0), phosphatidylcholine PC(12:0/13:0), phosphatidylethanolamine PE(17:0/17:0), phosphatidylglycerol PG(17:0/17:0), *d*_7_-cholesterol, sphingomyelin SM(d18:1/17:0), ceramide Cer(d18:1/17:0), monoacylglycerol MG(17:0/0:0/0:0), diacylglycerol DG(12:0/12:0/0:0) and triacylglycerol *d*_5_-TG(17:0/17:1/17:0), *d*_3_-palmitic acid], 750 μL of cold MTBE (methyl *tert*-butyl ether) containing the internal standard cholesteryl ester CE 22:1 and 188 μL of LC-MS grade water. After vortex mixing, samples were centrifuged at 14,000 × g for 2 min to separate the extracted phases. The upper hydrophobic fraction (350 µL) was collected for lipid analysis, and lower aqueous fraction (125 µL) was collected for metabolite analysis. Both fractions were evaporated to dryness using a Labconco CentriVap.

### Gas chromatography–time-of-flight mass spectrometry (GC–TOF MS) analysis for metabolites

GC–TOF MS analysis and data processing were performed as previously described^30^, using a Leco Pegasus IV time-of-flight mass spectrometer (Leco Corporation) coupled to an Agilent 6890 gas chromatograph (Agilent Technologies) equipped with an Rtx5Sil-MS column (30 m × 0.25 mm; 0.25 µm phase; Restek) and a Gerstel MPS2 automatic liner exchange system (Gerstel GMBH & Co. KG). Raw data files were processed using the metabolomics BinBase database^31^. All database entries in BinBase were matched against UC Davis metabolomics center’s mass spectral library.

### Reverse-phase lipid chromatography–quadrupole/time-of-flight mass spectrometry (CSH–QTOF MS) analysis for lipids

The lipid extracted phase was re-dissolved in a 90:10 methanol:toluene mixture (110 uL) (Fisher Scientific) containing 50 ng/mL CUDA (12-[[(cyclohexylamino)carbonyl]amino]- dodecanoic acid, Cayman Chemical) and analyzed using an Agilent 1290 Infinity LC system (Agilent Technologies). Analysis in both positive and negative ion modes and different mobile-phase modifiers for each polarity were used to increase the coverage of lipids measured^32^. For ESI(+) we used ammonium formate with formic acid as mobile phase modifiers. The addition of formic acid improved detection of CE, DG and PC lipid classes compared to ammonium formate alone. For ESI(−) we used ammonium acetate as mobile phase modifier. The volumes of 3 μL and 5 μL used for positive and negative ion modes respectively, were injected into an Acquity UPLC CSH C18 column (100 × 2.1 mm; 1.7 µm) coupled to an Acquity UPLC charged surface hybrid (CSH) C18 VanGuard pre-column (5 × 2.1 mm; 1.7 µm). The column was maintained at 65 °C with a flow-rate of 0.6 mL/min. Mobile phases were prepared with 10 mM ammonium formate and 0.1% formic acid for positive mode and 10 mM ammonium acetate for negative mode. Both positive and negative ion modes used the mobile phase composition of 60:40 acetonitrile:water (Fisher Scientific) for mobile phase A and 90:10 isopropanol:acetonitrile (Fisher Scientific) for mobile phase B. Gradient elution was performed from 0 min 15% (B), 0–2 min 30% (B), 2–2.5 min 48% (B), 2.5–11 min 82% (B), 11–11.5 min 99% (B), 11.5–12 min 99% (B), 12–12.1 min 15% (B), and 12.1–15 min 15% (B). Lipids were detected and quantified using an Agilent 6550 iFunnel accurate mass quadrupole/time-of-flight (QTOF) mass spectrometer with a jet stream ESI source (Agilent). The QTOF MS instrument was operated in electrospray ionization (ESI) in positive and negative mode with the following parameters: mass range, *m*/*z* 50–1700; capillary voltage, ±3 kV; nozzle voltage, ±1 kV; gas temperature, 200 °C; drying gas (nitrogen), 14 L/min; nebulizer gas (nitrogen), 35 psi; sheath gas temperature, 350 °C; sheath gas flow (nitrogen), 11 L/min; acquisition rate, 2 spectra/s. A reference solution (Agilent) was used to correct small mass drifts during the acquisition. Method blanks and human pooled plasma samples were used as QC controls. Quality control check showed that sample injection was not overloading the column (Figure S1). MS-DIAL software^33^ was used to process the raw data and lipids were reported only when detected in 50% of samples in each group. Annotations were made based on an in-house accurate mass and retention time lipid library created using LipidBlast, as described previously^34^.

### Statistical analyses

Univariate statistical analyses were implemented in R (v 3.2.4) statistical programming language and environment^35^. Mann-Whitney U test, which is more robust than T test when outliers are present or data violates the assumption of normality and heteroscedasticity, was performed to test for differences between all metabolite levels between the Control and HFHCC diet groups. The resulting test p-values were adjusted for the false discovery rate (FDR) using Benjamini–Hochberg procedure^36^ due to the multiple hypotheses tested, and reported as FDR-adjusted p values. Compared to the family-wise error rate correction (FWER) as described by Bonferroni, FDR adjustment is recommended when the number of tests in a study is large^37^. In this study, FDR-adjusted p values less than 0.05 were considered significant. For some metabolites of interest, nonadjusted p value was also reported although FDR-adjusted p value was not significant, in order to reduce the type II error rate (false negative).

### Principal component analyses (PCA) and heat map visualization

Peak heights of identified and unknown metabolites from GC–TOF MS and CSH–QTOF MS [from both ESI(+) and ESI(−) modes] were submitted using R to DeviumWeb^38^ for multivariate data analysis and visualization. GC-TOF MS results were mean-centered and divided by the standard deviation of each variable (center: mean; normalization: unit variance). PCA was used to identify clustering behavior related to different diets. For heat map generation, select metabolites from ESI(+/−) QTOF MS were visualized, showing significant differences between the two groups (adjusted p < 0.05). Fold change was calculated relative to the mean value of individual metabolite species within the control group.

## Results

### Non-obese NAFLD/NASH in HFHCC diet fed mice

**Figure 1:**
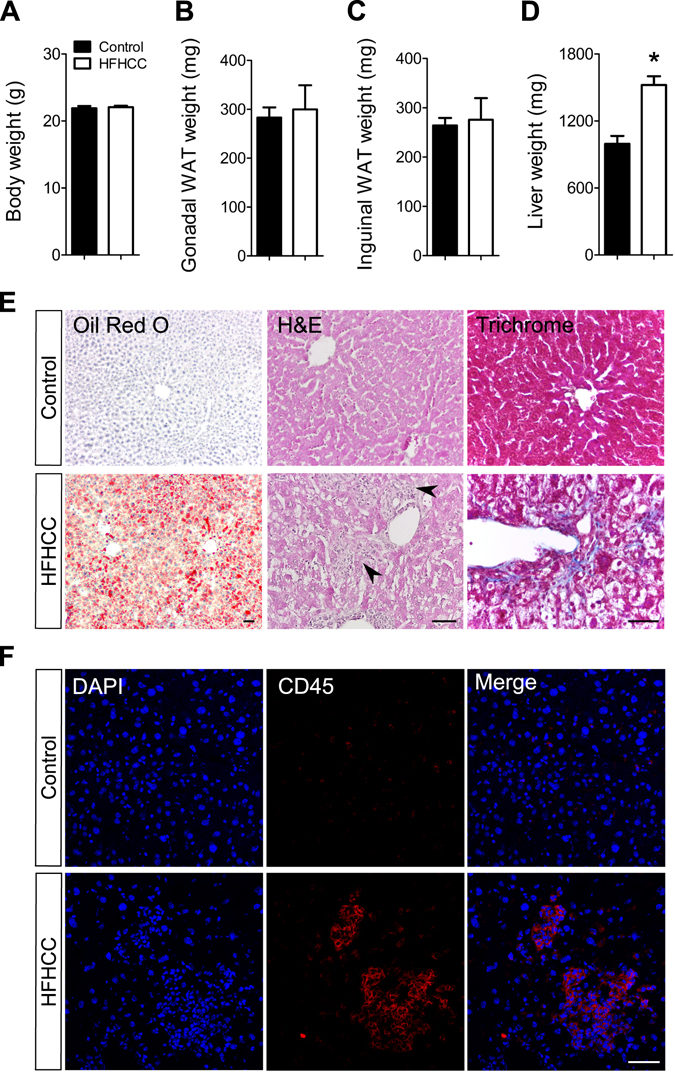
Weight parameters and hepatic pathology in HFHCC diet induced non-obese NAFLD/NASH. (**A**) Body weight was not significantly different between mice fed the HFHCC diet for three weeks compared to controls. (**B**,**C**) Weights of gonadal and inguinal white adipose tissues (WAT) were not significantly different between mice fed the HFHCC diet compared to controls. (**D**) Weights of livers from mice fed the HFHCC diet were significantly higher than controls (*p < 0.05). (**E**) Representative images showing neutral lipid staining using Oil Red O showed significant lipid accumulation (in red) only in the liver of HFHCC diet-fed mice. Histopathology examined after hematoxylin and eosin staining of liver sections showed prominent inflammatory loci (black arrows) only in the livers of HFHCC diet-fed mice. Trichrome staining detected collagen deposits (blue color) only in the livers of HFHCC diet-fed mice. (**F**) Immunohistochemical staining showed prevalence of CD45(+) immune cells in the livers of HFHCC diet-fed mice but not the control diet-fed mice. DAPI stained for nucleus. Scale bar: 50 µm (For all panels: n = 8 mice on Control diet and n = 6 mice on HFHCC diet were examined).

Mean body weight of mice fed the HFHCC diet for 3 weeks was not significantly different compared to mice on the Control diet (Fig. 1A). Mice on HFHCC diet consumed 2.74 g/day (3.8 kcal/g), and mice on Control diet consumed 3.27 g/day (3.3 kcal/g). Although feed consumption was lower with the HFHCC diet, total calories consumed per day were not different between the two groups (10.4 kcal/day for HFHCC and 10.8 kcal/day for Control diet). Analysis of body composition also indicated that mean weights of gonadal (visceral) and inguinal (subcutaneous) white adipose tissues (WATs) were not different between mice fed the HFHCC and Control diets (Fig. 1B and C). However, the HFHCC diet induced significant hepatomegaly as livers from HFHCC diet fed mice were ~50% heavier than those from the Controls (Fig. 1D). This confirmed that mice fed the HFHCC diet were not obese, but developed significant hepatic pathology. Liver sections stained for neutral lipids using Oil red O demonstrated prominent lipid accumulation in hepatocytes after HFHCC diet (Fig. 1E), which is the hallmark for hepatic steatosis. Histopathological examination of liver sections showed the presence of inflammatory loci in livers of mice fed HFHCC diet, but not in Control diet fed mice (Fig. 1E). Trichrome staining detected a minor degree of collagen deposits (blue color) only in livers of mice fed HFHCC diet, suggesting early mild fibrosis at this stage (Fig. 1E). To verify if inflammation is indeed active in livers of HFHCC diet-fed mice, we stained liver sections for CD45 antigen present in leukocytes. As expected, only livers of HFHCC-fed mice showed prominent staining of CD45 in the inflammatory loci detected by H&E staining, confirming infiltration of immune cells in the livers after HFHCC diet (Fig. 1F). These histopathological characteristics indicated that at 3 weeks of feeding HFHCC diet, mice undergo hepatic pathology indicative of NAFLD and progression to NASH.

### Distinct metabolite profiles in livers and plasma from HFHCC diet fed mice

**Figure 2:**
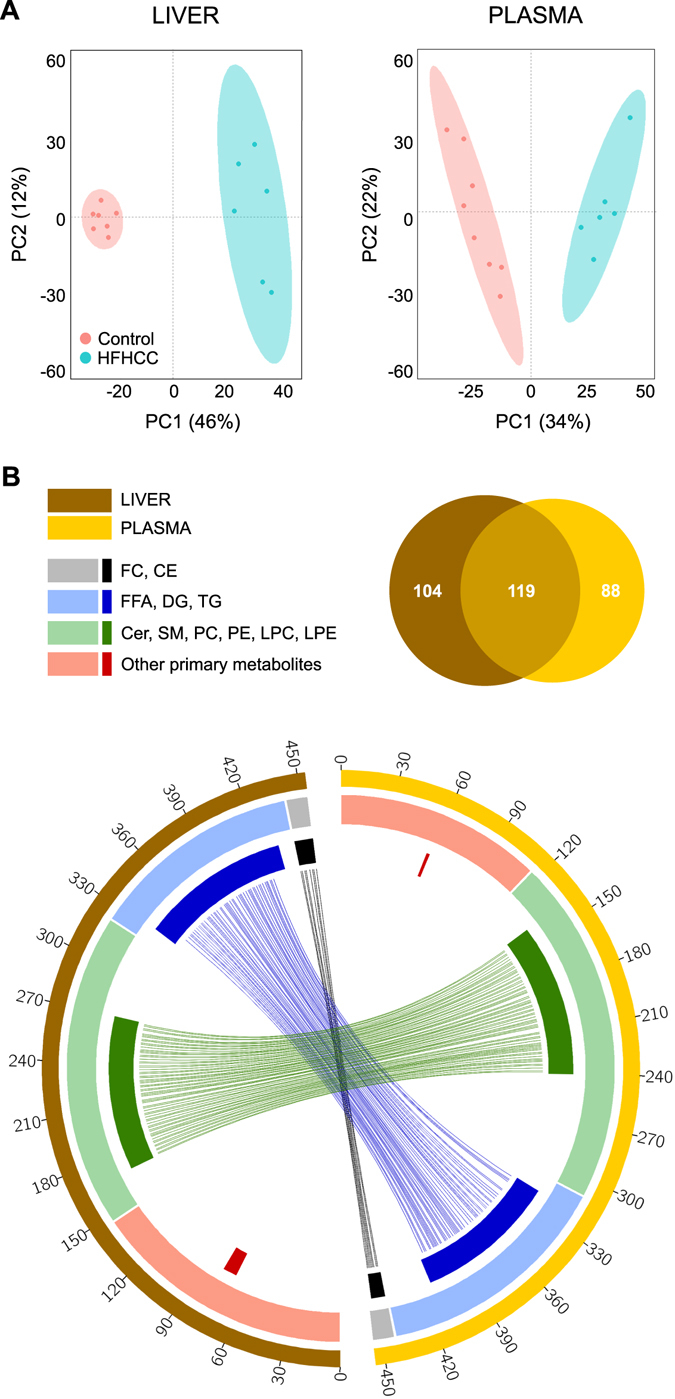
Metabolomics revealed global changes to liver and plasma metabolite composition in HFHCC diet-induced NAFLD/NASH. (**A**) Principal component analysis of liver and plasma samples from mice fed the HFHCC diet compared to controls showed distinct separations in clustering of metabolomes from the two diet groups. (**B**) Circos diagram showing relationship between the liver and plasma metabolomes after HFHCC diet induced non-obese NAFLD/NASH. Colored segments of the outer circle represent the number of metabolites identified in the liver and plasma of mice after the HFHCC diet. Colored segments of the middle circle represent different classes of metabolites. Colored sub-segments of the inner circle represent 223 genes and 207 genes significantly changed after the HFHCC diet in liver and plasma respectively (adjusted p < 0.05). Lines linking the segments indicate the 119 metabolites that were commonly altered in both liver and plasma of HFHCC diet-fed mice. Venn diagram shows the quantitative overlap between significantly different metabolites between liver and plasma samples after the HFHCC diet.

The profiles of metabolites identified in the livers and plasma from mice fed the HFHCC diet were distinct from the Control diet as observed by PCA (Fig. 2A). It was also evident that liver samples within the HFHCC diet group formed a loose cluster compared to liver samples from the Control diet group. CSH–QTOF MS and GC–TOF MS identified 223 metabolite species that were significantly altered in the liver after the HFHCC diet, with FDR-adjusted p value < 0.05, compared to Control diet (Fig. 2B). Similarly, 207 metabolite species were significantly different in the plasma after HFHCC diet, compared to the Control diet (Fig. 2B). Among the significantly different metabolite species in the livers and plasma, 119 were common. This high degree of overlap reflected a coherent relationship between the liver and plasma metabolite profiles. Besides, there was no difference in total levels of all lipid classes in livers of male and female mice fed HFHCC diet (Table S1 and S2).

### Cholesterol and bile acids were prominently elevated in HFHCC diet-induced NAFLD/NASH

**Figure 3:**
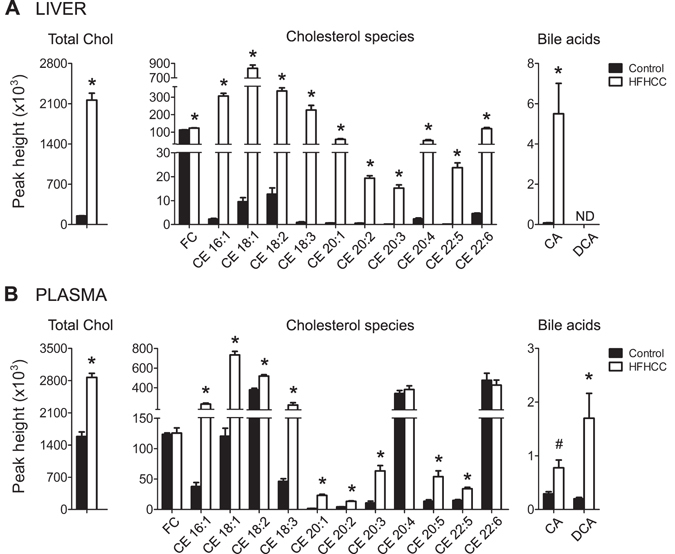
Cholesterol and bile acids were dramatically increased in HFHCC diet-induced NASH. (**A**) In the liver, total cholesterol was significantly increased in mice fed the HFHCC diet compared to controls. Free cholesterol (FC) was slightly but significantly elevated; all the different cholesteryl esters (CE) detected were markedly increased. The level of cholic acid (CA), the major bile acid formed in the liver was strikingly higher in mice fed the HFHCC diet, compared to the control group. (**B**) In the plasma, total cholesterol was again significantly increased in mice fed the HFHCC diet compared to controls. Plasma FC levels were not altered after HFHCC diet. All CE species, except CE 20:4 and 22:6, were significantly elevated in plasma from the HFHCC diet-fed mice compared to controls. Levels of CA and deoxycholic acid (DCA) after the HFHCC diet were markedly increased in plasma compared to controls (^#^p = 0.008/adjusted p = 0.12; *adjusted p < 0.05; n = 6 mice for HFHCC diet, n = 8 for Control diet).

Mice fed the HFHCC diet showed a marked increase in total cholesterol and all cholesteryl esters (CE) in the liver compared to Control diet (Fig. 3A). This HFHCC diet fed group also showed significant hypercholesterolemia and similar elevation of all CE species in the plasma except for CE 20:4 and CE 22:6 (Fig. 3B). The ester of oleic acid (CE 18:1) was the most abundant form of CE in both plasma and liver in the HFHCC diet as a result of a marked increase. This correlated with the increased level of free oleic acid by 3.8-fold in the livers of the HFHCC diet fed mice (not shown). Free cholesterol (FC) levels also showed a significant but modest increase in the livers of mice fed the HFHCC diet. FC levels were unchanged in the plasma after HFHCC diet compared to Control diet. Cholic acid (CA), the major bile acid, was significantly increased 68-fold in the liver and 2.6-fold in the plasma after HFHCC diet. High levels of CA could be derived from absorption of dietary cholate that is present in the HFHCC diet, but is also generated via conversion of cholesterol to CA as a mechanism to excrete excessive cholesterol. Deoxycholic acid (DCA), a byproduct of intestinal bacteria that metabolize dietary cholate, was significantly increased 8.5-fold in the plasma but was not detected in the liver of mice fed the HFHCC diet.

### FFA, DG and TG levels are altered in HFHCC diet-induced NAFLD/NASH

**Figure 4:**
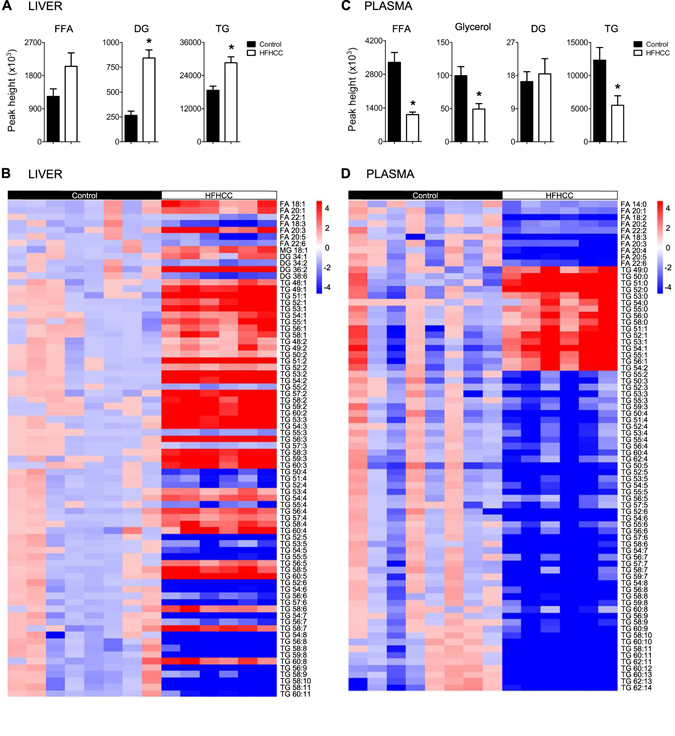
Lipid metabolism was significantly changed in mice fed the HFHCC diet. (**A**) In the liver, total free fatty acids (FFA) levels were elevated but not significant after HFHCC diet compared to controls. Total diacylglycerols (DG) and triacylglycerols (TG) levels were significantly increased after the HFHCC diet compared to controls. In absolute quantity, TG level was much more abundant than DG in the liver in both the HFHCC and control livers. (**B**) Analysis by constructing heat maps showed metabolite species that were significantly changed in the liver after the HFHCC diet. There was a trend that FFA, DG and TG that had higher degree of saturation (0–3 double bonds) were significantly increased, while those with higher degree of unsaturation (>3 double bonds) were significantly reduced in the liver after the HFHCC diet. (**C**) In the plasma, total FFA, glycerol and TG levels were significantly decreased in the HFHCC diet-fed mice compared to controls. DG levels were not different between the two groups. (**D**) Analysis by constructing heat maps showed metabolite species that were significantly changed in the plasma after the HFHCC diet. All FFA detected were significantly decreased in the HFHCC diet group. Saturated TG and monounsaturated TG were strongly elevated, whereas polyunsaturated TG (>2 double bonds) were significantly reduced. (*adjusted p < 0.05; n = 6 mice for HFHCC diet, n = 8 for Control diet).

Concurrent with cholesterol increases, diacylglycerols (DG) and triacylglycerols (TG) were significantly increased 3.17-fold and 1.53-fold respectively in the livers after HFHCC diet compared to Control diet (Fig. 4A). Levels of FFA were also elevated in HFHCC diet livers, but the values did not reach statistical significance when compared to Control diet. These combined findings indicate that lipid droplets seen in livers from HFHCC diet fed mice could be enriched with CE, TG and DG. When hierarchical cluster analysis was performed to visualize changes in subclasses of lipids (Fig. 4B), individual metabolite species within FFA, DG and TG showed both increases and decreases. Among the significantly different FFA, DG and TG species, the overall trend was that lipids containing low number of double bonds (0–3) were significantly increased whereas those with high number of double bonds (>3) were significantly decreased. This suggested an overall compositional change with increase in saturated and monounsaturated fatty acids, and a decrease in polyunsaturated fatty acids in DG and TG after HFHCC diet.

In plasma, total levels of FFA, glycerol and TG were all significantly reduced in HFHCC diet-fed group compared to the Control diet-fed mice (Fig. 4C). The levels of DG were unchanged between the two groups. Hierarchical cluster analysis demonstrated that all classes of FFA were lowered after HFHCC diet (Fig. 4D). The trend in the liver became more evident in the plasma that saturated and monounsaturated TG was highly elevated, while polyunsaturated TG (>2 double bonds) were significantly reduced in mice fed with HFHCC diet (Fig. 4D). The buildup of saturated and monounsaturated lipid species in both liver and plasma could be attributed to the composition of dietary fats presented to mice as cocoa butter^39^ in the HFHCC diet.

### Different classes of membrane phospholipids show distinct changes after HFHCC diet-induced NAFLD/NASH

**Figure 5:**
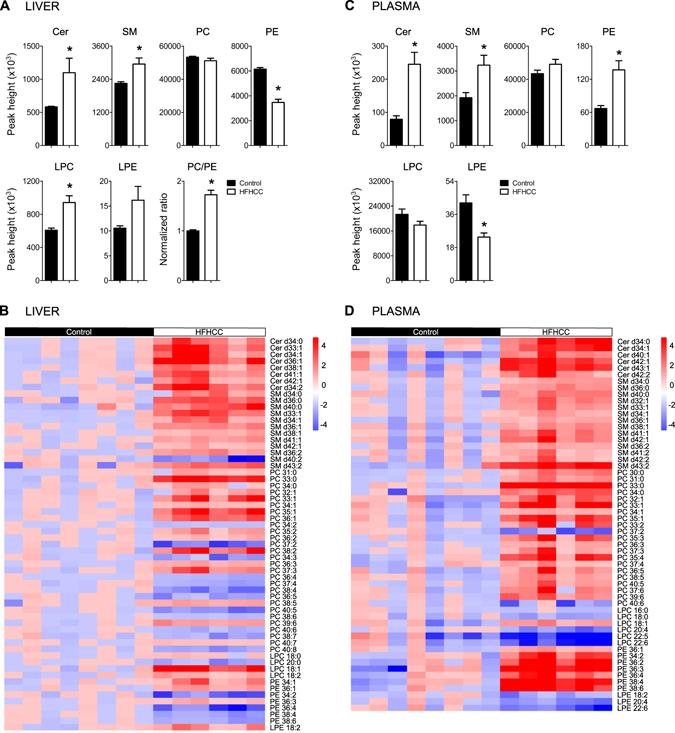
Phospholipid metabolism was significantly changed in mice fed the HFHCC diet. (**A**) In the liver, total ceramides (Cer) and sphingomyelins (SM) levels were both significantly elevated after HFHCC diet compared to controls. Total phosphotidylcholines (PC) levels remained unaffected, while total phosphoethanolamines (PE) levels were markedly reduced by 44% after the HFHCC diet compared to controls. Total lysophosphotidylcholines (LPC) levels were increased after HFHCC diet, and total lysophosphoethanolamines (LPE) levels were not significantly different. Hepatic PC/PE ratio was significantly higher in the HFHCC diet group compared to controls. (**B**) Analysis by constructing heat maps showed metabolite species that were significantly changed in the liver after HFHCC diet. All Cer and SM species, most of which were saturated and monounsaturated, were highly elevated. There was a trend that PC and PE species containing low number (0–3) of double bonds were elevated, while those with higher number (>3) of double bonds, were reduced. (**C**) In the plasma, total levels of Cer and SM were both significantly elevated in the HFHCC diet group. PC levels were unchanged, while PE levels were significantly increased in the HFHCC diet group compared to control diet group. LPC levels were unchanged, while LPE levels were significantly decreased in the HFHCC diet group compared to control diet group. (**D**) Analysis by constructing heat maps showed metabolite species that were significantly changed in the plasma after the HFHCC diet. All detected Cer, SM, PC and PE species were elevated, while LPC and LPE species appeared reduced. (*adjusted p < 0.05; n = 6 mice for HFHCC diet, n = 8 for Control diet).

Livers from mice fed the HFHCC diet had striking and consistent elevation of sphingophospholipids, which include ceramides (Cer; 1.88-fold) and sphingomyelins (SM; 1.31-fold), across all classes compared to controls (Fig. 5A and B). On examining glycerophospholipids, levels of phosphatidylcholines (PC) were not affected by the HFHCC diet in the liver compared to controls. However, levels of phosphatidylethanolamines (PE) were significantly reduced by 1.77-fold in the liver of the HFHCC diet-fed mice compared to controls. This dysregulation in PE raised the PC/PE ratio by 1.7-fold in the HFHCC diet-fed mice. The levels of glycerophospholipid metabolites, lysophosphotidylcholines (LPC) and lysophosphoethanolamines (LPE), were elevated in the liver but it was only significant for LPC. Comparisons of individual lipid species demonstrated a similar trend in FA compositions of liver PC and PE to that observed for DG and TG (above); saturated and monosaturated PC and PE showed significant increases, whereas polyunsaturated species were decreased (Fig. 5B).

In plasma samples from mice fed the HFHCC diet, elevation in Cer (3.1-fold) and SM (1.68-fold) was observed (Fig. 5C and D), similar to liver samples. Plasma levels of PC were not changed, and PE levels were markedly increased by more than 2-fold, a direction that was opposite to that observed in the liver. Also in contrast to the liver, plasma levels of LPC were not changed, and LPE levels were significantly reduced. Comparisons of individual lipid species demonstrated that regardless of saturation levels, almost all significantly changed PC and PE species were increased, whereas almost all LPC and LPE species were decreased in the plasma of HFHCC group compared to controls (Fig. 5D).

### Other primary metabolites in the liver altered in HFHCC diet-induced NAFLD/NASH

**Figure 6:**
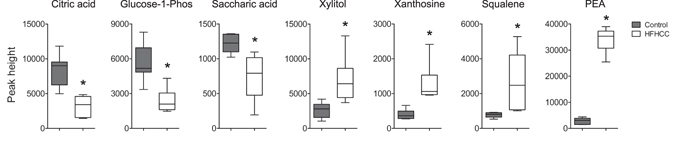
HFHCC diet altered levels of primary metabolites in the liver. Levels of citric acid, glucose-1-phosphate and saccharic acid were significantly reduced in mice after HFHCC diet compared to controls. Significant increases in xylitol, xanthosine, squalene and phenylethylamine (PEA) were observed after HFHCC diet compared to controls. (*adjusted p < 0.05; n = 6 mice for HFHCC diet, n = 8 for Control diet).

GC–TOF MS profiled primary metabolites including carbohydrates, amino acids, hydroxyl acids, FFAs, purines, pyrimidines and aromatics. Of the 146 and 115 primary metabolites detected in the liver and plasma samples respectively, excluding changes to bile acids and FFAs, only a few were significantly different between the HFHCC diet-fed mice and controls (Fig. 6). These metabolites were connected to glucose metabolism (glucose-1-phosphate, saccharic acid), tricarboxylic acid cycle (citric acid), xylose modification (xylitol), purine metabolism (xanthosine), a cholesterol precursor (squalene), and a trace amine (phenylethylamine/PEA).

## Discussion

Although metabolic syndrome is considered a major risk factor for NAFLD in patients, a non-obese sub-population without obvious risk factors also develops NAFLD. However, animal models that recapitulate the etiology and distinct pathophysiology of non-obese NAFLD remain to be defined. While studies have utilized diet-induced models like the methionine and choline-deficient (MCD) diet or the choline-deficient (CD) diet to indirectly affect fatty acid metabolism and induce the liver pathology associated with non-obese NAFLD^40^, it does not completely capture the contributing mechanisms of this condition in human patients. Several recent studies have renewed attention on high dietary cholesterol as a critical factor in non-obese NAFLD, and progression of NAFLD to NASH^21^. Moreover, studies have indicated that NAFLD/NASH is an independent risk factor for atherosclerosis^41–44^, suggesting common mechanistic elements underlying the etiology, and a sequential progression of the two pathologies. Therefore, the HFHCC diet that is also considered atherogenic with prolonged feeding uniquely captures early metabolic events of pathogenesis. In mice, it has been demonstrated that a HFHCC diet could induce experimental NAFLD/NASH, with histopathological similarities to human patients that are not observed in other models^26^. In this study, we investigate changes to the metabolic characteristics after HFHCC diet in mice, and document dietary cholesterol-mediated changes to understand non-obese NAFLD/NASH.

Pathogenesis of NAFLD in obese patients is marked by accumulation of TG, which are formed by FFA esterification within hepatocytes^45^. Insulin resistance in these individuals increases adipose tissue lipolysis causing a flux of FFA into plasma and then liver. The resulting steatosis in obese NAFLD is often associated with inflammation that can be due to several factors including the direct consequence of increased hepatic FFA^46, 47^. Excessive TG production is reflected in VLDL loading and hypertriglyceridemia. In contrast, the pathogenesis of non-obese NAFLD is distinct but understudied, with only a few reports on epidemiology and clinical cases. It has been indicated that NAFLD in non-obese patients presents with lower prevalence of diabetes and hypertriglyceridemia but an increased severity of inflammation even with a lower degree of steatosis and fibrosis^19^. In support of cholesterol as a factor, severity of inflammation in NAFLD has been associated with elevated FC levels^20^. In addition, FC levels have been directly linked to sensitivity towards cytokine-mediated steatosis^48^. In the HFHCC diet model, hepatic pathology is predominantly driven by cholesterol, and has been demonstrated to present a high level of inflammation^23^. Our results show that livers and plasma from HFHCC diet-fed mice have significant increases in FC and the different CEs in the liver. This is in contrast to MCD diet-fed mice in which cholesterol levels are not altered^49^, or in some cases have decreased^50^. Although liver FC levels progressively increase in obese NAFLD and NASH patients concurrent with TG, changes to CE levels are not observed^45^. In case reports on non-obese NAFLD patients, cholesterol levels appear to be moderately elevated, and in therapeutic intervention, suppressing cholesterol absorption improved liver dysfunction^51, 52^. Therefore, the predominant cholesterol-driven pathogenesis as seen in the HFHCC diet model could be mechanistically aligned with non-obese NAFLD in patients.

In support of this etiological distinction, mice fed the HFHCC diet showed a significant increase only in liver weight, without body weight gain or increases in fat pad weights indicating a metabolic distinction to obese NAFLD. Total cholesterol and total TG levels were elevated in the liver of HFHCC diet-fed mice. Despite significant hypercholesterolemia, plasma TG was significantly lower after HFHCC diet. These findings emphasize the critical nature of hepatic cholesterol-mediated dysregulation in the development of non-obese NAFLD/NASH. Plasma FFA and glycerol were also significantly reduced after HFHCC diet. These results could be interpreted as resulting from unaltered physiological insulin sensitivity and lower lipolysis occurring in adipose tissues, perhaps due to the rapid development of this non-obese NAFLD model. Concurrently, these observations indicated that the high fat content (37.1% of kcal) in the 3 weeks of HFHCC diet had only a less prominent role. We observed a marked increase in liver DG, suggesting that bioconversion of DG to TG may be dysregulated in HFHCC diet-fed mice. A previous study has shown that mice fed HFHCC diet downregulate liver acyl CoA: diacylglycerol acyltransferase (DGAT)^23^. Increases in hepatic DG levels have also been reported in human obese NAFLD patients and high fat diet mouse models^53^, this is mostly as a precursor for substantial TG biosynthesis that also elevate plasma TG levels, which was not observed in this HFHCC diet-induced NAFLD/NASH.

In more detailed interrogation, we identified a trend of increased saturated and monosaturated lipid species and decreased polysaturated lipid species in DG and TG in both liver and plasma after HFHCC diet-induced NAFLD/NASH. Although we suspect that this trend could be due to lipid species provided by cocoa butter^39^ in the HFHCC diet, it was similar to that reported in obese NAFLD patients^45^. The significance of this observation remains unclear.

Almost all SM species were dramatically increased in both liver and plasma after HFHCC diet, a phenomenon that has not been observed in obese NAFLD/NASH^45^. The plausible explanation for this increase is that FC could directly enhance *de novo* SM biosynthesis^54, 55^, which would also increase Cer as an intermediate. Cer to SM conversion by sphingomyelin synthase could also explain the consequent increase in DG levels^56, 57^, after the HFHCC diet. Based on the relationship between sphingomyelin synthase activity and cholesterol^58^, the increase in SM synthesis could be to facilitate a shift in homeostasis for elevated cholesterol metabolism. Nevertheless, these increases in SM and Cer could exacerbate hepatic pathology by increasing inflammation and apoptosis^59^, perhaps a significant secondary component of the pathologic basis of non-obese NAFLD/NASH. Moreover, the ensuing increase in SM and Cer in plasma suggests a shift in composition of circulating VLDL/LDL and could be exploited as a plasma marker for cholesterol-induced metabolic dysfunction and hepatic injury in non-obese NAFLD/NASH.

In experimental models, it is known that PC biosynthesis is linked to secretion of very low-density lipoproteins (VLDLs) from hepatocytes^60^. Reducing hepatic PC biosynthesis through a CD diet that decreases substrate for the CDP-choline pathway^60^, or by preventing conversion of PE to PC via deletion of phosphatidylethanolamine N-methyltransferase (PEMT)^61^, is known to cause a reduced PC/PE ratio and steatosis. In contrast, PC levels after the HFHCC diet were not different in both the liver and plasma; however, PE levels were markedly reduced in the livers resulting in an increased PC/PE ratio. Potentially explaining this observation, a previous study reported that mice fed HFHCC diet have a 10-fold upregulation of choline kinase^23^, the first enzyme in the CDP-choline pathway, suggesting that a shift in balance to *de novo* PC synthesis rather than conversion of PE to PC. Moreover, LPC was significantly upregulated in the liver in mice fed HFHCC diet suggesting a higher rate of PC to LPC conversion. Although the role of PE in defining VLDL secretion and composition is not completely understood, there is evidence that maintenance of membrane fluidity under conditions of elevated FC would need a decrease in PE levels^62^. Plasma concentrations of PE are much lower than PC, and the absolute value albeit significantly increased after the HFHCC diet was merely a fraction compared to values observed for PC.

In obese NAFLD/NASH patients, the relationship between the PC/PE ratio and severity of steatosis and inflammation is not evident. For example, a lipidomic investigation in obese NAFLD and NASH patients showed that both PC and PE levels significantly decreased in obese NAFLD livers, but were unchanged in obese NASH livers compared to physiological controls^45^. Another similar study showed that changes to PC and PE levels in human obese NAFLD/NASH patients are very modest compared to a high-fat diet mouse model^53^. Our findings after HFHCC diet clearly demonstrate an increase in hepatic PC/PE ratio. Although it is unknown whether this has a link to pathologic severity, this unique characteristic could be an indicator for cholesterol-induced non-obese NAFLD/NASH.

Together with cholesterol, it has been demonstrated that the ratio of plasma LDL/VLDL increases by a remarkable 120 folds after three weeks of the HFHCC diet^23^. Moreover, altered hepatic SM metabolism after increased dietary cholesterol has also been shown to increase the levels of SM in the VLDL fraction^63^. Increased SM would facilitate cholesterol loading during VLDL maturation, introducing a homeostatic element to this compositional shift. In addition, this compositional shift in phospholipids could explain unchanged levels of plasma PC after the HFHCC diet.

**Figure 7:**
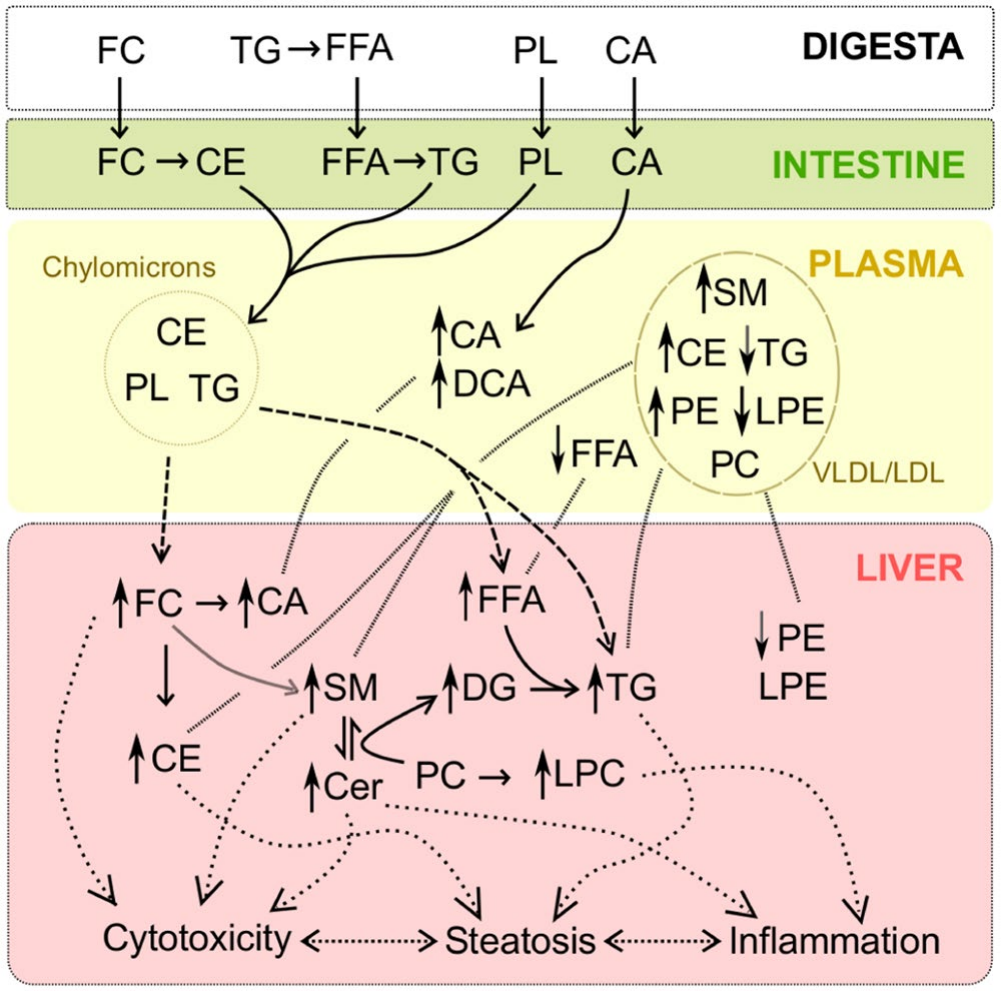
Model: Mechanistic reconstruction of metabolic changes as observed in the HFHCC diet-induced NAFLD/NASH. High abundance of dietary cholesterol initiated a lipid imbalance in the liver and also the plasma. Presence of dietary CA facilitated lipid absorption and contributed to the severity of the phenotype in the short 3 weeks of the HFHCC diet. High levels of FC and CE in the liver affected SM metabolism. This resulted in the significant increase in levels of hepatic SM, Cer and DG. Levels of hepatic TG were also elevated due to sequestration from plasma, with only a putative minor contribution from the elevated FFA and DG levels. Levels of PC were not affected both in the liver and plasma, whereas PE levels showed a decrease in the liver and an increase in the plasma. A compositional shift of VLDL/LDL could explain the increased plasma CE, SM and PE levels. High hepatic levels of CE and TG were the contributing factors for steatosis. High hepatic levels of FC, SM, Cer and FFA were the contributing factors for cytotoxicity and inflammation leading to steatohepatitis.

Hepatotoxicity of cholesterol has been reasonably established^64^, and could be the core primary component of hepatic pathology induced by the HFHCC diet. Presence of cholate in this diet complements this pathogenesis not only by enhancing lipid absorption in the intestine, but also by enhancing inflammation in the liver when in excess^65^. In agreement, bile acids levels have been reported to be elevated in patients with NASH^66, 67^. The different changes to *in vivo* metabolism in HFHCC diet induced NAFLD/NASH (Fig. 7), reveals a mechanism distinct from NAFLD associated with the metabolic syndrome.

Only a few changes to primary metabolites between control and HFHCC diet in both liver and plasma confirmed that lipids primarily drove this metabolic pathology. The 7 metabolites that were significantly different were not restricted to a specific pathway indicating that effects, if any, were not robust. Moreover, it is possible that significant elevation of squalene and PEA could be due to enrichment in the dietary components and not relevant to non-obese NAFLD/NASH. However, metabolomics data from the understudied non-obese NAFLD/NASH patients are not currently available to make direct comparisons and examine the finer points of this *in vivo* murine model.

In summary, this study sets the stage in describing for the first time a metabolic phenotype that has almost all the hallmarks of non-obese NAFLD/NASH in human patients. Our results demonstrate mechanisms distinct from that reported for other NAFLD/NASH murine models, and may provide valuable insights into diagnosis and management of non-obese NAFLD/NASH in human patients.

## Electronic supplementary material

Supplementary Information
