# Supplementary material for: Metabolomic characteristics of cholesterol-induced non-obese nonalcoholic fatty liver disease in mice
Source: Sci Rep. 2017 Jul 21;7:6120. doi: 10.1038/s41598-017-05040-6 (PMC5522413; doi:10.1038/s41598-017-05040-6)
Supplement: Supplementary file 1 — Supplementary Information [file 41598_2017_5040_MOESM1_ESM.pdf]

Metabolomic characteristics of cholesterol-induced non-obese nonalcoholic fatty liver disease in mice

Lan N. Tu[1], Megan R. Showalter[2], Tomas Cajka[2], Sili Fan[2], Viju V. Pillai[1], Oliver Fiehn[2,3], Vimal Selvaraj[1,§]

Supplementary Information

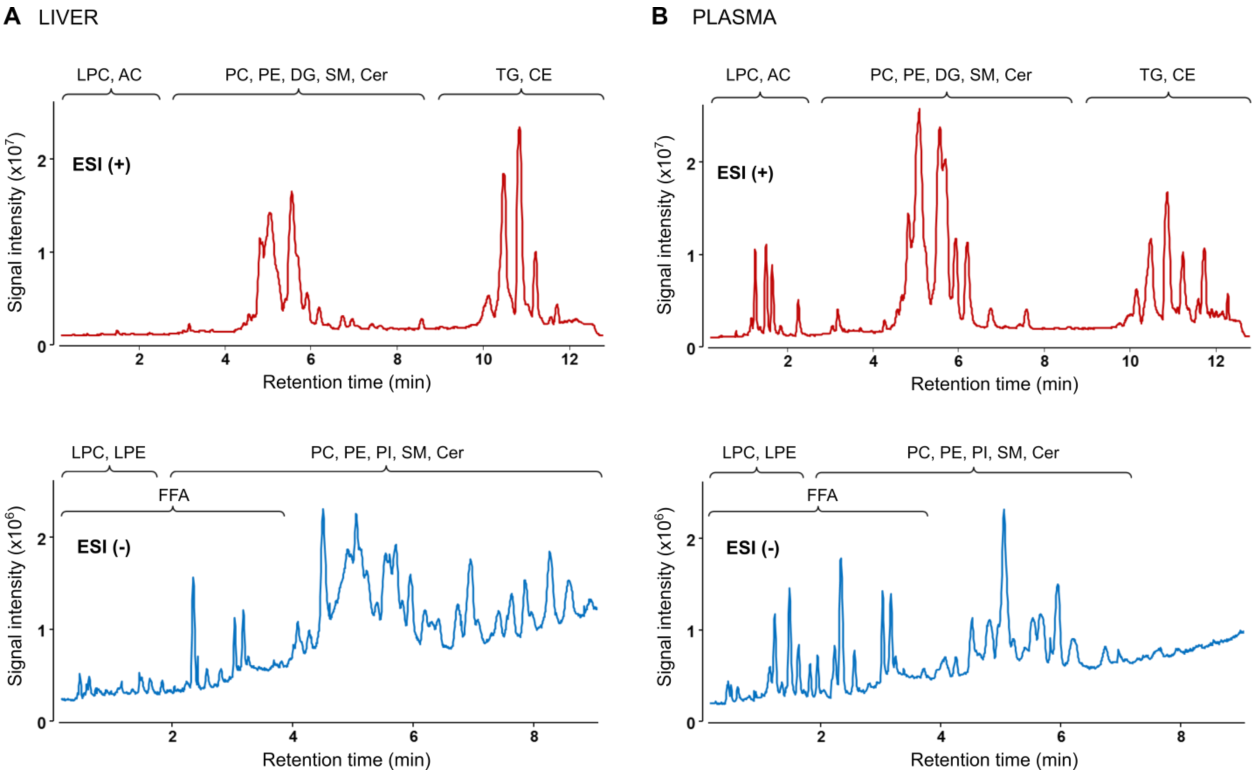

Figure S1. Representative total ion chromatograms for plasma and liver lipids. Quality control check of all samples showed that sample injection was not overloading the columns. Elution of particular lipid classes for each electrospray ionization (ESI) mode was highlighted by retention time ranges. Data from a representative liver (A) and plasma (B) sample from an animal fed with HFHCC diet were shown. The followings are indicated: acylcarnitines (AC), cholesteryl esters (CE), diacylglycerols (DG), triacylglycerols (TG), lysophosphatidylcholines (LPC), lysophosphatidylethanolamines (LPE), phosphatidylcholines (PC), phosphatidylethanolamines (PE), ceramides (Cer), sphingomyelins (SM), free fatty acids (FFA), phosphatidylinositols (PI).

Table S1. Total levels of different lipid classes in livers of male and female mice after HFHCC diet.

Lipid class	Male (n=3)	Female (n=3)	P value
	Peak height (x10^3)		
Cholesterol	1975.2 ± 167.7	2351.2 ± 90.5	0.120
FFA	2605.5 ± 383.8	1492.1 ± 463.0	0.138
DG	862.4 ± 59.3	823.4 ± 169.9	0.839
TG	26955.6 ± 3563.1	30267.4 ± 2858.8	0.500
Cer	1493.0 ± 275.6	703.4 ± 88.9	0.053
SM	3076.4 ± 413.6	2823.6 ± 244.8	0.627
PC	52959.3 ± 2618.3	49369.1 ± 1120.6	0.276
PE	3898.1 ± 307.4	3040.6 ± 225.1	0.088
LPC	1086.6 ± 46.5	799.6 ± 101.7	0.062
LPE	20.8 ± 2.6	11.6 ± 3.3	0.095

Table S2. Total levels of different lipid classes in plasma of male and female mice after HFHCC diet.

Lipid class	Male (n=3)	Female (n=3)	P value
	Peak height (x10^3)		
Cholesterol	2966.8 ± 128.3	2773.6 ± 116.8	0.328
FFA	1148.1 ± 213.4	1097.0 ± 112.9	0.843
DG	21.5 ± 6.6	15.4 ± 5.8	0.529
TG	5013.9 ± 1488.0	6004.5 ± 2805.5	0.771
Cer	288.0 ± 67.2	202.7 ± 9.3	0.277
SM	3686.4 ± 746.7	2785.7 ± 150.9	0.303
PC	52833.8 ± 5080.1	45293.3 ± 1828.8	0.235
PE	157.1 ± 30.1	116.8 ± 8.3	0.266
LPC	19235.0 ± 2149.8	16505.5 ± 982.8	0.312
LPE	26.1 ± 4.3	21.2 ± 0.7	0.327
